# CTX-M-27 Producing *Salmonella enterica* Serotypes Typhimurium and Indiana Are Prevalent among Food-Producing Animals in China

**DOI:** 10.3389/fmicb.2016.00436

**Published:** 2016-03-31

**Authors:** Wen-Hui Zhang, Xiang-Yan Lin, Liang Xu, Xi-Xi Gu, Ling Yang, Wan Li, Si-Qi Ren, Ya-Hong Liu, Zhen-Ling Zeng, Hong-Xia Jiang

**Affiliations:** National Risk Assessment Laboratory for Antimicrobial Resistance of Animal Original Bacteria, College of Veterinary Medicine, South China Agricultural UniversityGuangzhou, China

**Keywords:** *Salmonella*, food-producing animals, serotype, antimicrobial resistance, CTX-M

## Abstract

*Salmonella* spp. is one of the most important food-borne pathogens causing digestive tract and invasive infections in both humans and animals. Extended-spectrum β-lactamases (ESBLs) especially the CTX-M-type ESBLs are increasingly being reported worldwide and in China. These studies seldom focused on *Salmonella* isolates from food-producing animals. The aim of this study was to characterize the antimicrobial resistance profiles, serotypes and ESBLs and in particular, CTX-M producing *Salmonella* isolates from chickens and pigs in China. *Salmonella* isolates were identified by API20E system and polymerase chain reaction (PCR) assay; serotypes were determined using slide agglutination with hyperimmune sera; antimicrobial susceptibility was tested using the ager dilution method; the prevalence of ESBLs and PMQR genes were screened by PCR; CTX-M-producing isolates were further characterized by conjugation along with genetic relatedness and plasmid replicon type. In total, 159 *Salmonella* strains were identified, among which 95 strains were *Salmonella enterica* serovar Typhimurium, 63 strains were *S. enterica* serovar Indiana, and 1 strain was *S. enterica* serovar Enteritidis. All of these isolates presented multi-drug resistant phenotypes. Forty-five isolates carried *bla*_CTX-M_ genes, the most common subtype was CTX-M-27(34), followed by CTX-M-65(7) and CTX-M-14(4). Most *bla*_CTX-M_ genes were transmitted by non-typeable or IncN/IncFIB/IncP/IncA/C/IncHI2 plasmids with sizes ranging from 80 to 280 kb. In particular, all the 14 non-typeable plasmids were carrying *bla*_CTX-M-27_ gene and had a similar size. PFGE profiles indicated that CTX-M-positive isolates were clonally related among the same serotype, whilst the isolates of different serotypes were genetically divergent. This suggested that both clonal spread of resistant strains and horizontal transmission of the resistance plasmids contributed to the dissemination of *bla*_CTX-M-9G_-positive *Salmonella* isolates. The presence and spread of CTX-M, especially the CTX-M-27 in *S. enterica* serovars Typhimurium and Indiana from food-producing animals poses a potential threat for public health. Control strategies to limit the dissemination of these strains through the food chain are necessary.

## Introduction

The emergence of antibiotic-resistant bacteria has become a serious challenge in human and veterinary medicine globally and poses a serious public health threat. Non-typhoidal *Salmonella* (NTS) are a leading cause of bacterial diarrhea worldwide and one of the most important foodborne pathogens. The majorities of human infections by NTS are associated with food product consumption of meat, eggs, milk, seafood, and other fresh products derived from animals (Foley and Lynne, 2008). In the animal husbandry industry, antibiotics are used both for therapy and as growth promoters. Additionally, animals are always a large repository of resistant bacteria. Domestic farm animals, especially poultry and pigs have been shown to be major environmental reservoirs of food-borne NTS (Vo et al., 2006).

Serotyping is a useful classification scheme that allows for trends in *Salmonella* surveillance data to be followed over time. These patterns are also related to the ability to cause disease and with the associated antibiotic resistance profile. Over 2,600 serotypes of *Salmonella* have been identified based on the reactivity of antisera to O and H antigens (Stevens et al., 2009), and there are numerous overlaps between animal and human *Salmonella* serotypes (Alcaine et al., 2006). This suggests that *Salmonella* transmission from animals to humans occurs via the food chain. *Salmonella enterica* serovars Enteritidis and Typhimurium are the most common serotypes in Europe (European Centre for Disease Prevention and Control, 2013). However, serotype distribution is varied in different provinces of China and in total the most prevalent serovars were also Typhimurium and Enteritidis with a rising rate of reports of *S. enterica* serovar Indiana in isolates obtained from animals (Xia et al., 2009; Yang et al., 2010; Lu et al., 2014; Zhang et al., 2014).

It is estimated that that between 1994 and 2005 roughly 22% of foodborne diseases in China were caused by *Salmonella* though there was no official surveillance data (Wang et al., 2007). In 2013, unpublished data from the China CDC surveillance system showed that the carriage rate of human salmonellosis was 549 per 100,000 people. This is more than 33 times higher than human infections in the USA in 2012 (16.4 per 100,000; Centers for Disease Control and Prevention, 2013; Sun et al., 2014). There is also an increasing risk of *Salmonella* spread from animals to humans *via* the food chain due to worldwide distribution of animal food products from China. Extended spectrum cephalosporins (ESCs) and fluoroquinolones are the drugs of choice for treatment of invasive *Salmonella* infections in food animals and for people at risk of such infections (Harrois et al., 2014). This is the focus of current concerns on the emergence of resistance to these drugs. ESCs- and fluoroquinolone-resistant *Salmonella* populations have increased dramatically worldwide (Arlet et al., 2006; Hendriksen et al., 2009; Jiang et al., 2014; Lu et al., 2014). Resistance to cephalosporins is mainly due to the acquisition of extended-spectrum β-lactamases (ESBLs) genes especially the CTX-M type that is primarily carried by transferable plasmids and transposons (Liebana et al., 2013). These plasmids and transposons, in many cases, also carry resistance genes for other antimicrobial classes such as the fluoroquinolones which sometimes limits treatment options (Harrois et al., 2014). Plasmid-mediated quinolone resistance (PMQR) has recently been categorized as containing three distinct resistance mechanisms: (1) Qnr proteins that mediate target protection, (2) A variant of an aminoglycoside acetyltransferase designated AAC(6’)-Ib-cr that acetylates ciprofloxacin and norfloxacin and (3) Drug eﬄux mediated by OqxAB and QepA (Strahilevitz et al., 2009). Although these PMQR determinants confer only low-level fluoroquinolone resistance, their presence can provide a selective advantage for bacteria to develop to high-level quinolone resistance. This occurs by chromosomal mutations in the quinolone resistance-determining regions (QRDRs) of genes encoding target enzymes (Strahilevitz et al., 2009). The situation is especially troubling when ESBL genes and PMQR genes are co-transmitted through transferable plasmids.

Indiscriminate use of antibiotics in both human and veterinary medicine make the ESBLs especially the CTX-M-producing bacteria including *Salmonella* increase rapidly accelerating the spread of antibiotic resistance. There have been several reports regarding characteristics of CTX-M-producing *Salmonella* isolated from food-producing animals in China. However, data on distribution of serotypes and antimicrobial susceptibility of *Salmonella* recovered from animals are available in most of these reports (Yan et al., 2010; Yang et al., 2010; Li R. et al., 2013; Li et al., 2014; Lu et al., 2014; Kuang et al., 2015). The aim of the present study was to investigate the prevalence of antibiotic resistance, serotypes and ESBLs among *Salmonella* isolates from chickens and pigs in China. Plasmids that carried *bla*_CTX-M_ genes were also further studied to characterize the mechanism of transfer and dissemination of β-lactamases.

## Materials and Methods

### *Salmonella* Isolation, Identification, and Serotyping

In 2014, a total of 3850 non-repetitive fecal swabs were collected from healthy chickens and pigs in Guangdong, Shandong, Henan, Hubei provinces of China. Of these samples, 2090 samples were from chickens and 1760 samples were from pigs.

Cotton swabs of feces were inoculated into sterile selenite cystine broth for 24 h at 37°C, and then streaked onto chromogenic medium selective for *Salmonella* (CHROMagar Microbiology, France) and incubated for another 24 h at 37°C. One purple colony was selected from each plate and then confirmed using the API20E system (bioMérieux, Marcy l’Étoile, France) and a PCR assay targeting the *invA* gene (Rahn et al., 1992). *Salmonella* isolates were serotyped using slide agglutination with hyperimmune sera (S&A Company, Bangkok, Thailand) and the results were interpreted according to the Kauffmann–White scheme. All identified isolates were stored at –80°C in Luria–Bertani (LB) broth containing 30% glycerol.

### Antimicrobial Susceptibility Testing

The minimum inhibitory concentrations (MICs) of *Salmonella* isolates were determined using the agar dilution method following Clinical and Laboratory Standards Institution guidelines. Sixteen antimicrobials were tested: ampicillin (AMP), cefotaxime (CTX), cefoxitin (CXT), ceftiofur (CTF), ceftazidime (CAZ), ceftriaxone (CTR), nalidixic acid (NAL), ciprofloxacin (CIP), enrofloxacin (ENR), kanamycin (KAN), gentamycin (GEN), amikacin (AMK), tetracycline (TET), chloramphenicol (CHL), florfenicol (FFC) and olaquindox (OLA). The results were interpreted according to the standards described by CLSI (M100-S25) (Clinical and Laboratory Standards Institute [CLSI], 2015) (ampicillin, cefotaxime, ceftiofur, ceftriaxone, cefoxitin, ceftazidime, nalidixic acid, ciprofloxacin, kanamycin, amikacin) and VET01-A4/VET01-S2 (Clinical and Laboratory Standards Institute [CLSI], 2013) (gentamicin, enrofloxacin, tetracycline, chloramphenicol, florfenicol) and the DANMAP 98 (olaquindox). *Escherichia coli* ATCC 25922 was used as quality control.

### Detection of β-Lactamase Genes and PMQR Genes

The *Salmonella* isolates harvested from Mueller Hinton (MH) agar plates supplied with 2 mg/L cefotaxime were subjected to screening for ESBL-genes using PCR as described previously (Jiang et al., 2012). The DNA sequences and deduced amino acid sequences were compared with the reported sequences from GenBank^1^. The presence of PMQR determinants [*qnrA, qnrB, qnrS, qnrD, qnrC, aac(6’)-Ib-cr,qepA*, and *oqxAB*] was also analyzed using PCR primers and conditions described previously (Jiang et al., 2012). Mutations in QRDR of target genes (*gyrA* and *parC*) were further analyzed in both PMQR and CTX-M-positive isolates as described previously (O’Regan et al., 2009).

### Molecular Typing

Genetic relatedness of all *bla*_CTX-M_ -positive isolates was analyzed by pulsed-field gel electrophoresis (PFGE) after *Xba*l-digested genomic DNA using a CHEF-MAPPER System (Bio-Rad Laboratories, Hercules, CA, USA) as described previously (Jiang et al., 2014). The resulting PFGE patterns were compared using the Dice similarity coefficient of BioNumerics software (Applied Maths, Sint-Martens-Latem, Belgium).

### Conjugation and Transformation Experiments

Conjugation experiments were conducted in *bla*_CTX-M_-positive strains by liquid mating-out assay in LB-medium using a sodium azide-resistant *E. coli* J53 as the recipient. Transconjugants were selected on MacConkey agar containing cefotaxime (2 mg/L) and sodium azide (300 mg/L). For transformation experiments, plasmid DNA from the *bla*_CTX-M_-positive strains were extracted using Qiagen Plasmid Midi Kit according to the manufacturer’s instructions (Qiagen, Germany). Purified plasmids were transformed into *E. coli* DH5a (TaKaRa Biotechnology, Dalian, China). Selection of transformants was performed on MacConkey agar containing 2 mg/L cefotaxime. MICs and the presence of *bla*_CTX-M_ gene of transconjugants and transformants were determined as described above.

### Plasmid Characterization

PCR-based replicon typing (PBRT) was performed on transconjugants and transformants using primers as described previously (Carattoli et al., 2005). Then PFGE with S1 nuclease (Takara Biotechnology, Dalian, China) digestion of whole genomic DNA was carried out as described previously (Barton et al., 1995). The resulting gels were analyzed by Southern transfer and probing with a DIG-labeled *bla*_CTX-M_ gene fragment according to the manufacturer’s instructions (DIG High Prime DNA Labeling and Detection Starter Kit I, Roche Applied Science, Mannheim, Germany).

## Results

### *Salmonella* Isolation, Identification, and Serotyping

A total of 159 *Salmonella* strains were obtained among which 90 (56.6%) were isolated from pigs and 69 (43.4%) were recovered from chickens. In all, three serotypes were identified in the 159 *Salmonella* isolates and this accounted for 95 strains of *S. enterica s*erovar Typhimurium, 63 strains of *S. enterica* serovar Indiana, and 1 strain of *S. enterica* serovar Enteritidis.

The *S. enterica s*erovar Typhimurium isolates were distributed as follows: 73.7% from pigs and 26.3% from chickens while the Indiana isolates were almost evenly distributed (50.8% from chickens and 49.2% from pigs, relatively).

### Antimicrobial Susceptibility Phenotypes

Among the159 *Salmonella* isolates, resistance was most frequently observed to tetracycline (89.9%), ampicillin (84.9%), and gentamicin (81.1%). Resistance rates of the quinolone antibiotics were all above 50% including resistance to nalidixic acid (77.3%), ciprofloxacin (64.8%), and enrofloxacin (62.9%). Cephalosporin resistance rates ranged from 17.6 to 76.7% with high resistance rates to ceftriaxone (76.7%), ceftiofur (52.8%), and cefotaxime (47.2%). Resistance rates to ceftazidime and cefoxitin were 30.8 and 17.6%, respectively. The presence of resistance to olaquindox, a growth promoter used extensively in pigs and recently prohibited for poultry use, was detected in 65.4% of all isolates (**Table 1**).

**Table 1:** Antimicrobial resistance of *Salmonella enterica* isolates from food-producing animals in China.

Antimicrobial agents	Number(%) of resistant isolates (*n* = 159)
**β-Lactams**	
Ampicillin	135 (84.9)
Cefotaxime	75 (47.2)
Cefoxitin	28 (17.6)
Ceftiofur	84 (52.8)
Ceftazidime	49 (30.8)
Ceftriaxone	122 (76.7)
**Quinolones**	
Nalidixic acid	123 (77.4)
Ciprofloxacin	103 (64.8)
Enrofloxacin	100 (62.9)
Aminoglycosides	
Kanamycin	124 (78.0)
Gentamicin	129 (81.1)
Amikacin	9 (5.7)
**Other Antibiotics**	
Tetracycline	143 (89.9)
Chloramphenicol	126 (79.2)
Florfenicol	124 (78.0)
Olaquindox	104 (65.4)

All the 159 *Salmonella* isolates were multi-drug resistant displaying resistance to three or more classes of antimicrobials. Higher resistance frequencies were found among *S. enterica s*erovar Indiana isolates compared with *S. enterica s*erovar Typhimurium isolates. For example, cefotaxime resistance in Indiana and Typhimurium isolates were 71.4 and 30.5%, respectively, while the ciprofloxacin resistance rates were 73.0 and 43.2%, respectively (**Table 2**).

**Table 2:** Antibiotic resistance rate of *Salmonella enterica* serotype Typhimurium and Indiana isolates (%).

Serotype Antibiotics	AMP	CTF	CTX	CXT	CTZ	CTR	GEN	KAN	AMI	CIP	ENR	NAL	TET	CHL	FFC	OQX
Indiana (63)	93.7	76.2	71.4	19.0	33.3	82.5	88.9	84.1	6.3	73.0	77.8	87.3	96.8	90.5	84.1	68.3
Typhimurium (95)	89.5	37.9	30.5	11.6	29.5	73.7	76.8	74.7	5.3	60.0	52.6	70.5	86.3	72.6	74.7	64.2

### ESBL Characterization

Seventy-five isolates displayed non-wild type MICs for cefotaxime and 45 of these carried *bla*_CTX-M_ genes, three harbored *bla*_CMY-2_ one of which coexisted with the CTX-M-encoding gene. For other β-lactamase genes, narrow-spectrum β-lactamase *bla*_TEM-1_ was present in 86 of the 159 isolates, *bla*_SHV-1_ and *bla*_OXA-1_ genes were found in 62 and 55 of these isolates, respectively. No other type of ESBLs gene was found.

For the PMQR genes, three classes of resistance mechanisms were represented: *oqxAB* (78.0%, *n* = 124); *aac(6’)-Ib-cr*(69.2%, *n* = 110) and *qnr(*17.6%, *n* = 28). For 28 *qnr* genes, 12 were *qnrD*, 7 were *qnrB*, 5 were *qnrS*, and the remaining 4 were *qnrA.* All *qnr*-positive strains also carried *oqxAB* and/or *aac(6’)-Ib-cr.* At least one PMQR gene was found in each CTX-M-producing strains, especially the *aac(6’)-Ib-cr* +*oqxAB* and qnr+*aac(6’)-Ib-cr* +*oqxAB* were found in 17 and 4 of all 45 CTX-M-producing strains, respectively. Mutations in the QRDRs of *gyrA* and *parC* were analyzed among the 45 PMQR and CTX-M-positive isolates. A combination of mutations in *gyrA*83 (S83F) and 87 (D87G) and in *parC* 80(S80R) were found in 35 (77.8%) isolates, a signal mutation in *gyrA* 87 (D87N) without mutation in *parC* was detected in the remaining 10 isolates (**Table 3**).

**Table 3:** Characteristics of CTX-M-producing *Salmonella enterica* isolates from food-producing animals in China.

Strain	Source	Serotype	ESBL gene	PMQR	Amino acid substitutions		Co-transferred resistant gene	Plasmid replicon type	Plasmid approx. size(kb)
					*gyrA*	*parC*			
SP5	Pig	Typhimurium	*bla*_CTX-M-14_	*aac-(6’)-Ib-cr,oqxAB*	D87N	–	*bla*_SHV -1_, *aac-(6’)-Ib-cr*	FIB	100
SP85	Pig	Indiana	*bla*_CTX-M-27_	*aac-(6’)-Ib-cr oqxAB*	S83F,D87G	S80R	*bla*_OXA-1_,*aac-(6’)-Ib-cr*	NT	100
SP96	Pig	Indiana	*bla_CTX-M-27_*	*oqxAB*	S83F,D87G	S80R	*bla*_SHV -1_, *oqxAB*	P	150
SP99	Pig	Typhimurium	*bla_CTX-M-27_*	*qnrB, aac-(6’)-Ib-cr,oqxAB*	S83F,D87G	S80R	*aac-(6’)-Ib-cr oqxAB*	NT	100
SP101	Pig	Typhimurium	*bla_CTX-M-65_*	*oqxAB*	D87N	–	*oqxAB*	FIB	150
SP108	Pig	Indiana	*bla_CTX-M-27_*	*aac-(6’)-Ib-cr,oqxAB*	S83F,D87G	S80R	*bla*_SHV -1_, *aac-(6’)-Ib-cr*	A/C	150
SP110	Pig	Indiana	*bla_CTX-M-65_*	*oqxAB*	S83F,D87G	S80R	*bla_SHV -1_*	N	100
SP115	Pig	Indiana	*bla_CTX-M-27_*	*aac-(6’)-Ib-cr,oqxAB*	S83F,D87G	S80R	*aac-(6’)-Ib-cr*	NT	100
SP123	Pig	Indiana	*bla_CTX-M-27_*	*oqxAB*	D87N	–	*–*	FIB	150
SP125	Pig	Indiana	*bla_CTX-M-27_*	*oqxAB*	D87N	–	*–*	FIB	150
SP129	Pig	Typhimurium	*bla_CTX-M-27_*	*aac-(6’)-Ib-cr,oqxAB*	S83F,D87G	S80R	*bla_SHV -1_,aac-(6’)-Ib-cr*	P	80
SP131	Pig	Indiana	*bla_CTX-M-65_*	*aac-(6’)-Ib-cr,oqxAB*	S83F,D87G	S80R	*bla_SHV -1,_ aac-(6’)-Ib-cr*	N	150
SP132	Pig	Indiana	*bla_CTX-M-27_*	*oqxAB*	S83F,D87G	S80R	*bla_SHV -1,_ oqxAB*	N	150
SP2019	Pig	Typhimurium	*bla_CTX-M-14_*	*aac-(6’)-Ib-cr,oqxAB*	D87N	–	*bla_OXA-1,_ oqxAB*	HI2	280
CL108	Chicken	Indiana	*bla_CTX-M-27_*	*oqxAB*	S83F,D87G	–	*–*	NT	100
CL129	Chicken	Indiana	*bla_CTX-M-27_*	*aac-(6’)-Ib-cr*	S83F,D87G	S80R	*bla_SHV -1,_aac-(6’)-Ib-cr*	FIB	150
CL135	Chicken	Indiana	*bla_CTX-M-27_*	*oqxAB*	S83F,D87G	S80R	*–*	NT	100
CL140	Chicken	Indiana	*bla_CTX-M-27_*	*aac-(6’)-Ib-cr*	S83F,D87G	S80R	*–*	NT	100
CL146	Chicken	Indiana	*bla_CTX-M-27_*	*qnrD,aac-(6’)-Ib-cr,oqxAB*	S83F,D87G	S80R	*bla_SHV -1,_aac-(6’)-Ib-cr*	NT	100
CL189	Chicken	Indiana	*bla_CTX-M-27_*	*oqxAB*	S83F,D87G	S80R	*oqxAB*	HI2	200
XC48	Chicken	Typhimurium	*bla_CTX-M-27_*	*aac-(6’)-Ib-cr, oqxAB*	S83F,D87G	S80R	*aac-(6’)-Ib-cr*	N	150
XC87	Chicken	Typhimurium	*bla_CTX-M-65_*	*aac-(6’)-Ib-cr,oqxAB*	S83F,D87G	S80R	*aac-(6’)-Ib-cr oqxAB*	N,	150
XC134	Chicken	Typhimurium	*bla_CTX-M-14_*	*aac-(6’)-Ib-cr,oqxAB*	S83F,D87G	S80R	*oqxAB*	N,A/C	150
XC164	Chicken	Typhimurium	*bla_CTX-M-27_*	*oqxAB*	S83F,D87G	S80R	*bla_TEM-1b,_oqxAB*	HI2	194
HB73	Chicken	Indiana	*bla_CTX-M-27_*	*oqxAB*	S83F,D87G	S80R	*–*	A/C	130
HB137	Chicken	Indiana	*bla_CTX-M-27_*	*aac-(6’)-Ib-cr,oqxAB*	S83F,D87G	S80R	*oqxAB*	NT	100
HB154	Chicken	Indiana	*bla_CTX-M-65_*	*aac-(6’)-Ib-cr,oqxAB*	S83F,D87G	S80R	*aac-(6’)-Ib-cr oqxAB*	N,	130
SG36	Chicken	Indiana	*bla_CTX-M-27_*	*oqxAB*	S83F,D87G	S80R	*bla*_SHV -1_	NT	100
SG119	Chicken	Indiana	*bla_CTX-M-27_*	*aac-(6’)-Ib-cr*	S83F,D87G	S80R	*–*	NT	100
MM62	Chicken	Enteritidis	*bla_CTX-M-27_*	*aac-(6’)-Ib-cr*	D87N	–	*aac-(6’)-Ib-cr*	P	80
PY12	Chicken	Indiana	*bla_CTX-M-14_*	*aac-(6’)-Ib-cr,oqxAB*	D87N	–	*bla_SHV -1_*	N,A/C	130
K13	Chicken	Indiana	*bla_CTX-M-27_*	*aac-(6’)-Ib-cr,oqxAB*	S83F,D87G	S80R	*–*	NT	100
K14	Chicken	Indiana	*bla_CTX-M-27_*	*oqxAB*	S83F,D87G	S80R	*–*	NT	100
K17	Chicken	Typhimurium	*bla_CTX-M-27_*	*aac-(6’)-Ib-cr,oqxAB*	S83F,D87G	S80R	*aac-(6’)-Ib-cr*	NT	100
K19	Chicken	Indiana	*bla_CTX-M-65_*	*aac-(6’)-Ib-cr,oqxAB*	S83F,D87G	S80R	*aac-(6’)-Ib-cr,oqxAB*	N	150
K21	Chicken	Indiana	*bla_CTX-M-27_*	*aac-(6’)-Ib-cr*	D87N	–	*aac-(6’)-Ib-cr*	FIB	150
K46	Chicken	Indiana	*bla_CTX-M-27_*	*aac-(6’)-Ib-cr,oqxAB*	S83F,D87G	S80R	*aac-(6’)-Ib-cr,oqxAB*	P	100
K47	Chicken	Indiana	*bla_CTX-M-27_*	*aac-(6’)-Ib-cr*	S83F,D87G	S80R	*aac-(6’)-Ib-cr*	NT	100

*NT, non-typeable incompatibility (Inc) group plasmid. The underline isolates were transconjugants, the others were transformants.*

A total of three different CTX-M ESBLs were detected in these isolates and they all belonged to the CTX-M-9 group with the CTX-M-encoding genes distributed as 34 *bla*_CTX-M-27_, 7 *bla*_CTX-M-65_, and 4 *bla*_CTX-M-14_. The distribution of these three CTX-M subtypes showed no specificity between serotypes and animals and were found in both Indiana and Typhimurium isolates from pigs and chickens. Antimicrobial susceptibility testing demonstrated that CTX-M-27-positive isolates had significantly higher resistance levels than the CTX-M-14 or CTX-M-65 -positive isolates.

### Plasmid Characterization

Six transconjugants and 32 transformants were successfully obtained from 45 *bla*_CTX-M_-positive isolates by conjugation/transformation experiments with frequencies of 10^-8^ to 10^-3^ per donor cell. PMQR genes were found to be co-transferred with *bla*_CTX-M_ genes in 26 (68.4%) transconjugants/transformants and the most common co-transferring pattern was *bla*_CTX-M_ +*aac(6’)-Ib-cr* accounting for 13 of 26 transconjugants/transformants, *bla*_CTX-M_ +*oqxAB* and *bla*_CTX-M_ +*aac(6’)-Ib-c*r +*oqxAB* accounting for eight and five transconjugants/transformants, respectively. For narrow-spectrum β-lactamases, *bla*_SHV -1_, *bla*_OXA-1_, and *bla*_Tem-1b_ were co-transferred with *bla*_CTX-M_ in 11, 2, and 1 isolates, respectively.

Plasmid replicon typing of transconjugants/transformants revealed that IncN, IncFIB, IncP, IncA/C, and IncHI2 were detected in 9, 6, 4, 4, and 3 transconjugants/transformants, respectively. Two plasmid replicons (IncN in combination with IncA/C) were simultaneously present in 2 CTX-M-14-producing transformants. Surprisingly, the replicon types could not be determined by PBRT in 14 transconjugants/transformants all of which carried the *bla*_CTX-M-27_ gene (**Table 3**).

Southern blot hybridization suggested that the plasmid sizes of *bla*_CTX-M_ genes varied between 80 and 280 kb among which the 100 kb (*n* = 17) and 150 kb (*n* = 13) were the most common sizes. Interestingly, the size of all the 14 non-typeable *bla*_CTX-M-27_ plasmids was ∼100 kb.

### Pulsed-Field Gel Electrophoresis (PFGE)

**FIGURE 1:**
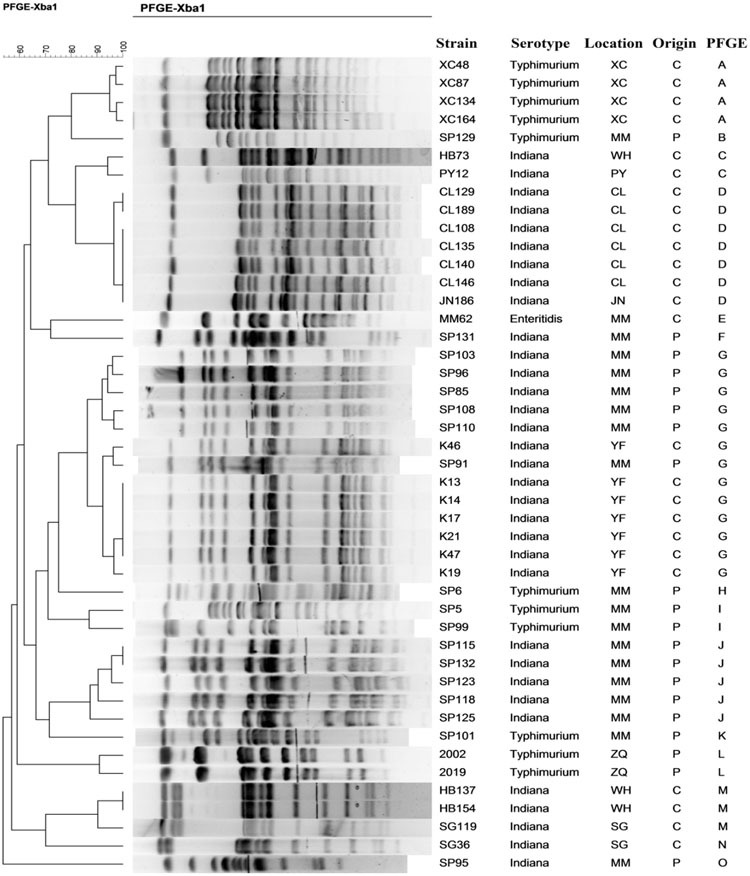
**Pulsed-field gel electrophoresis fingerprinting patterns of *Xba* I-digested total DNA preparations from *Salmonella isolates* harboring CTX-M-encoding genes**.

A total of 34 different PFGE profiles were obtained from the 45 *bla*_CTX-M_-positive isolates (**Figure 1**). These could be grouped into 15 PFGE clusters designated A–O having 85% genetic similarity. Clusters G, D, J, and A accounted for 28.9, 15.6, 11.1, and 8.9% of the isolates, respectively. In addition, all isolates in each cluster had the same serotype and were also isolated from the same province (same or different cities). The exceptions were clusters C and M both of which were comprised of two isolates each from different provinces (Hubei, Henan, or Shan dong provinces).

In the most predominant cluster G, a high similarity of PFGE profiles of the isolates could be found and the isolates were recovered from pigs or chickens in two cities of Guangdong province. Seven isolates in this cluster were recovered from chickens and six were from pigs. In the remaining 14 PFGE clusters, isolates were all recovered from the same origin; either chickens or pigs.

## Discussion

In this study, we examined *Salmonella* isolates recovered from food-producing animals to determine their serotypes, antimicrobial susceptibility phenotypes, and genotypes. The results showed that *S. enterica* serovars Typhimurium and Indiana were the prevailing serotypes of *Salmonella* in pigs and chickens. Both serovars were resistant to multiple antimicrobials including ESCs and fluoroquinolones which are often antimicrobials of choice for treating salmonellosis. Resistance to cefotaxime could be explained by the presence of non-typeable and IncN/IncFIB/IncP/IncA/C/IncHI2 plasmids carrying *bla*_CTX-M-9G_ genes. The high detection rate of PMQR determinants especially *aac(6’)-Ib-c*r and *oqxAB* and mutations in QRDR of target genes made an important contribution to the non-wild type MICs of the fluoroquinolones.

*Salmonella* has various serotypes, and some serotypes may also relate to multidrug-resistant phenotypes (Meunier et al., 2002). The predominant serotypes change over time and differ from one geographical area to another. Additionally, a particular serovar can prevail and emerge within a region for a certain period while being completely absent in other regions. *S. enterica* serovar Indiana is rarely reported worldwide but there have been large-scale food poisoning outbreaks caused by this serotype in both the USA and Europe (Price and Carter, 1967; Campbell and Eckman, 1975). In China, little information about *S. enterica* serovar Indiana was available until this serotype was obtained from a food animal (Yan et al., 2010) and showed resistance to multiple antimicrobial agents. Following this, isolation of *S. enterica* serovar Indiana increased rapidly and it became more and more prevalent in China especially in isolates from veterinary clinics and animal foods. The characteristics of multidrug-resistant Indiana in China were also reported (Xia et al., 2009; Yang et al., 2010; Lu et al., 2014). In the present study, all of the 159 *Salmonella* isolates displayed resistance to three or more classes of antimicrobials, but *s*erovar Indiana isolates showed higher resistance levels than *s*erovar Typhimurium isolates. Our results indicate that the multi-resistant serotype Indiana along with Typhimurium isolates are of high prevalence among food-producing animals in China.

CTX-M type ESBLs have been widely reported globally for many years and there is a strong linkage between their emergence and increasing quinolone resistance in the Enterobacteriaceae (Lavilla et al., 2008). The co-existence or co-transfer of PMQR genes in ESBLs and especially in CTX-M-producing isolates has been also previously described (Liu et al., 2013; Jiang et al., 2014; Li et al., 2014; Yang et al., 2015). These may promote the development of multidrug-resistant isolates under selective pressure of the quinolone and/or cephalosporin (Liu et al., 2013). We found all CTX-M-encoding strains contained at least one PMQR genes especially the *aac(6’)-Ib-cr and oqxAB* genes. These two PMQR genes have been shown to accelerate the development of fluoroquinolone resistance in *S. typhimurium* (Wong et al., 2014a) and were frequently co-transferred with *bla*_CTX-M_ genes. Previous reports showed that *aac(6’)-Ib-cr* and *oqxAB* were also prevalent PMQR genes in *E. coli* isolates from humans, animals and the environment (Chen et al., 2012). In general, detection rates of PMQR genes in *Salmonella* were less than those observed in *E. coli*. However, these rates in *Salmonella* increased quickly in China especially *aac(6’)-Ib-cr* and *oqxAB.* In the present study, 78.0 and 69.2% harbored *oqxAB* and *aac(6’)-Ib-cr*, which is higher than levels previously reported in 2009–2010 (32.3%, 25.8%), 2007–2011(30.8%, 30.8%) and in 2012–2013 (31.7%, 36.5%) in China (Li et al., 2013; Jiang et al., 2014; Li et al., 2014). Our results may indicate that ESBL genes along with PMQR genes are increasing among *Salmonella* strains in China.

The CTX-M type β-lactamases are the most prevalent ESBLs among the Enterobacteriaceae recovered from both animals and humans worldwide (D’Andrea et al., 2013). The spread and characteristics of CTX-M type ESBLs producing pathogenic bacteria had been widely studied previously in China, but works seldom focused on food-borne *Salmonella*. In the present study, 60% of cefotaxime-resistant isolates produced CTX-M type ESBLs and three different CTX-M-9 group variants were detected among which the CTX-M-27 enzyme was the most dominant. Consistent with the results in this study, the CTX-M-27 enzyme was also the main type of ESBL in our previous study with *Salmonella* (Jiang et al., 2014) and we also previously found the CTX-M-27 was the only CTX-M-type ESBLs detected in *Salmonella* isolates of retail pork (unpublished data). However, only two reports were found about CTX-M-27-producing human clinical *Salmonella* strains. One study showed that CTX-M-27-producing isolates of *S. enterica* serotype Livingstone were the cause of a nosocomial outbreak in the neonatal ward, the other study showed *bla*_CTX-M-27_ along with *oqxAB* on the IncHI2 plasmid which was similar to that of the strain CL189 in our study (Bouallegue-Godet et al., 2005; Wong et al., 2014b). Many reports suggested that CTX-M-14 and CTX-M-55 enzymes were the two most dominant types in *E. coli* obtained from food animals in China (Zheng et al., 2012; Rao et al., 2014). CTX-M-27 in *E. coli* was only sporadically detected in several reports (Liu et al., 2007; Lu et al., 2010; Ho et al., 2011; Ma et al., 2012; Zheng et al., 2012; Rao et al., 2014) among which two studies found its high presence in *E. coli* isolated from duck and environmental samples and aquatic sediment samples (Lu et al., 2010; Ma et al., 2012). Study on isolates from human beings and environmental samples suggested that most CTX-M-27-positive *E. coli* from different hospitals and from rivers and lakes belonged to the O25b-ST131-B2 or B2:ST131 which was strongly associated with potentially severe infections in both humans and animals (Matsumura et al., 2012; Zurfluh et al., 2013; Micenkova et al., 2014). The high prevalence of *bla*_CTX-M-27_ in *Salmonella* obtained from food animals and in *E. coli* isolates from different sources (human beings, environment, and animals) should be a cause for concern which may be a potential threat to public health.

CTX-M-27 which was first found in a clinical isolate from a French hospital differed from its ancestor-CTX-M-14 only by the substitution D240G, but had significant higher hydrolytic activity against ceftazidime (Bonnet et al., 2003). Previous studies (Rao et al., 2014) suggested that CTX-M-1G-positive isolates had significantly higher resistance rates to cefquinome, ceftazidime, amikacin and fosfomycin when compared to the CTX-M-9G-positive isolates. These authors also compared the activity of different CTX-M variants on cephalosporin resistance and proved that CTX-M-1 group enzymes, especially CTX-M-55, have higher hydrolytic activities against cefquinome than CTX-M-9 group enzymes (except CTX-M-27). All the *bla*_CTX-M_ genes detected in this study belonged to CTX-M-9 group. When we analyzed the MICs of cephalosporin among different CTX-M-9G variants, we concluded that *bla*_CTX-M-27_-positive isolates had a higher MIC for cephalosporin in particular to ceftazidime than that in CTX-M-14 and CTX-M-65-producing isolates. There were few differences in the resistance phenotypes of different variants of the CTX-M-9G family of β-lactamases isolates. This may also explain the prevalence of different variants.

In this study, 38 *bla*_CTX-M_-positive transconjugants/transformants were obtained, among which 68.4% co-transferred *bla*_CTX-M_ with *aac(6’)-Ib-cr* and/or *oqxAB*, indicating that these fluoroquinolone resistant determinants can be transferred horizontally simultaneous with the transfer of CTX-M-encoding genes. Characterization of *bla*_CTX-M_ plasmids in the transconjugants/transformants revealed that *bla*_CTX-M_ genes were located on plasmids of un-typeable and IncN/IncFIB/IncP/IncA/C/IncHI2 replicon types with different plasmid sizes. In particular, we observed that plasmids carrying *bla*_CTX-M-27_ from epidemiologically unrelated strains (from different geographic regions and sources) had surprisingly similar properties (replicon types and sizes) indicating the presence of epidemic *bla*_CTX-M-27_ plasmids in China. It’s worth noting that the replicon types were untypeable using the current PBRT scheme in half (14/28) of the transconjugants/transformants carrying *bla*_CTX-M-27_. Previous studies on *bla*_CTX-M-27_ also found some plasmids could not be determined by PBRT (Ma et al., 2012). Therefore, there may be a new type of plasmid-carrying *bla*_CTX-M-27_. We have sequenced two *bla*_CTX-M-27_ plasmids from different sources to further study their characteristics (unpublished data).

Among the five detected plasmid replicons in present study, the IncP type plasmid carrying *bla*_CTX-M_ in *Salmonella* isolates was reported for the first time. In general, most *bla*_CTX-M_ genes can be horizontally transferred. Untypeable plasmids and IncN/IncFIB/IncP/IncA/C/IncHI2 plasmids can drive such increased dissemination in *Salmonella* isolates from food animals. To the best of our knowledge, the current study is also the first report about the spread of non-typeable or IncN/IncFIB/IncP/IncA/C/IncHI2 plasmids carrying *bla*_CTX-M_ genes in *S. enterica* serovars Typhimurium and Indiana isolated from pigs and chickens in China.

PFGE is a gold-standard for *Salmonella* subtyping and has been widely used to determine the relatedness of pathogens to confirm the outbreaks and trace the source of the isolates (White et al., 2007). Fifteen different PFGE clusters (85% genetic similarity) were obtained in this study and these represented a wide variety of genotypes. However, isolates recovered from the same or even different provinces in each identified serovars were highly genetically similar (cluster A, D, G, J and cluster C, M). These may have derived from a specific clone, so the horizontal transmission of the resistance plasmids along with clonal spread of resistant strains were both responsible for dissemination of *bla*_CTX-M-9G_-positive strains.

All of the 45 *bla*_CTX-M-9G_-positive *Salmonella* strains were isolated from food animals from different provinces. Therefore, humans can be infected through the consumption of food of animal origin (Thorns, 2000), accelerating the transmission of such *Salmonella* strains. Along with this, food animals trading may be an important source of *Salmonella* colonization as has been previously assumed (Wegener et al., 2003). Though studies proving definitive evidence of ESC-R bacterial transmission from food animals to humans are limited; differences can always be shown when comparing *Salmonella* isolates from humans and the animals. Importantly, previous studies have shown that the same IncN plasmids harboring *bla*_CTX-M_ are simultaneously present in isolates from pigs and farm workers (Moodley and Guardabassi, 2009). Therefore, further study monitoring and comparing antimicrobial susceptibility, serotypes and ESBL-producing *Salmonella* isolates from both veterinary settings (pets, wild animals, and livestock) and humans (communities and hospitals) in the same geographic region could lead to the discovery of dissemination routes.

## Conclusion

Multi-drug resistant *S. enterica* serotype Typhimurium and Indiana and especially CTX-M-producing isolates were predominant among food-producing animals in China. Untypeable and IncN/IncFIB/IncP/IncA/C/IncHI2 resistant plasmids harboring *bla*_CTX-M_ genes and clonal spread of strains were both responsible for dissemination of resistant *Salmonella* isolates. The presence and spread of *bla*_CTX-M_, especially the *bla*_CTX-M-27_ on non-typeable plasmids in *Salmonella* isolates from food-producing animals may pose a potential threat for public health. Proper molecular approaches and hygienic practices are urgently needed to control the dissemination of resistant *Salmonella* strains through food chain. As ceftiofur and cefquinome have been widely used as therapeutic antibiotic in veterinary medicine, the third- and fourth-generation cephalosporins should be used more prudently in animal husbandry in China.

## Author Contributions

Conceived and designed the experiments: Z-LZ, H-XJ, and Y-HL. Performed the experiments: W-HZ, X-YL, LX, and X-XG. Analyzed the data: W-HZ and X-YL. Contributed reagents/materials/analysis tools: LY, WL, and S-QR. Wrote the paper: W-HZ and H-XJ.

## Conflict of Interest Statement

The authors declare that the research was conducted in the absence of any commercial or financial relationships that could be construed as a potential conflict of interest.

## Footnotes

1 www.ncbi.nlm.nih.gov/genbank/
